# Gain/Amplification of Chromosome Arm 1q21 in Multiple Myeloma

**DOI:** 10.3390/cancers13020256

**Published:** 2021-01-12

**Authors:** Ichiro Hanamura

**Affiliations:** Division of Hematology, Department of Internal Medicine, Aichi Medical University School of Medicine, 1-1, Karimata, Yazako, Nagakute, Aichi 480-1195, Japan; hanamura@aichi-med-u.ac.jp; Tel.: +81-561-62-3311 (ext. 23450); Fax: +81-561-63-3401

**Keywords:** multiple myeloma, 1q21, chromosomal instability of 1q12, *CKS1B*, *MCL1*

## Abstract

**Simple Summary:**

Multiple myeloma (MM), a plasma cell neoplasm, is an incurable hematological malignancy. Gain/amplification of chromosome arm 1q21 (1q21+) is the most common adverse genomic abnormality associated with disease progression and drug resistance. While possible mechanisms of 1q21+ occurrence and candidate genes in the 1q21 amplicon have been suggested, the precise pathogenesis of MM with 1q21+ is unknown. Herein, we review the current knowledge about the clinicopathological features of 1q21+ MM, which can assist in effective therapeutic approaches for MM patients with 1q21+.

**Abstract:**

Multiple myeloma (MM), a plasma cell neoplasm, is an incurable hematological malignancy characterized by complex genetic and prognostic heterogeneity. Gain or amplification of chromosome arm 1q21 (1q21+) is the most frequent adverse chromosomal aberration in MM, occurring in 40% of patients at diagnosis. It occurs in a subclone of the tumor as a secondary genomic event and is more amplified as the tumor progresses and a risk factor for the progression from smoldering multiple myeloma to MM. It can be divided into either 1q21 gain (3 copies) or 1q21 amplification (≥4 copies), and it has been suggested that the prognosis is worse in cases of amplification than gain. Trisomy of chromosome 1, jumping whole-arm translocations of chromosome1q, and tandem duplications lead to 1q21+ suggesting that its occurrence is not consistent at the genomic level. Many studies have reported that genes associated with the malignant phenotype of MM are situated on the 1q21 amplicon, including *CKS1B*, *PSMD4*, *MCL1*, *ANP32E*, and others. In this paper, we review the current knowledge regarding the clinical features, prognostic implications, and the speculated pathology of 1q21+ in MM, which can provide clues for an effective treatment approach to MM patients with 1q21+.

## 1. Introduction

Multiple myeloma (MM) is a plasma cell neoplasm accounting for around 10% of hematological malignancies [1]. MM is thought to develop through a multistep process, including genomic instability, epigenetic dysregulation, and interactions with the bone marrow niche during which clonal evolution occurs, and a normal B-cell goes through the stages of monoclonal gammopathy of uncertain significance (MGUS) to smoldering MM (SMM) [2]. At the cytogenetic level, transformation from normal late-stage B-cells to malignant plasma cells (MM cells) is due to primary *IGH* (immunoglobulin heavy chain) translocations [3,4,5] or hyperdiploidy, which are mutually exclusive in most cases [6,7,8,9].

**Figure 1:**
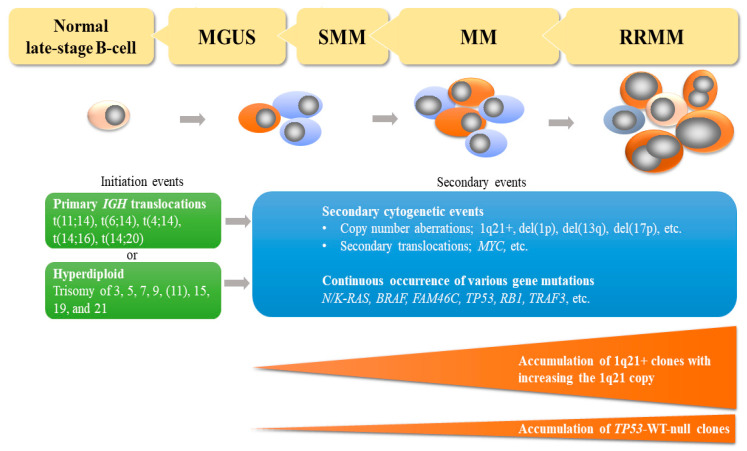
Genomic abnormalities in development of multiple myeloma. Multiple myeloma (MM) develops from a normal late-stage B-cell through monoclonal gammopathy of undetermined significance (MGUS) and smoldering myeloma (SMM). Incidence of gain or amplification of chromosome arm 1q21 (1q21+) increases from MGUS to relapsed MM, which is from less than 20% to about 70%. Incidence of the biallelic inactivation of *TP53* also increase at relapse. RRMM, relapsed and/or refractory multiple myeloma; IGH, immunoglobulin heavy chain; del(1p), deletion of chromosome arm 1p; del(13q), deletion of chromosome arm 13q; del(17p), deletion of chromosome arm 17p; WT, wild type.

The major *IGH* translocations are t(4;14), t(6;14), t(11;14), t(14;16), and t(14;20) leading to the overexpression of the genes *MMSET* and *FGFR3* (about 70% of patients with t(4;14)), *CCND3*, *CCND1*, *MAF*, and *MAFB*, respectively [10,11,12,13,14]. The aberrant expressions are due to dysregulation by the regulatory elements of the *IGH* gene. Hyperdiploidy is characterized by multiple trisomies of chromosomes 3, 5, 7, 9, 11, 15, 17, 19, and 21 [7]. Secondary genomic events such as chromosomal copy number abnormalities other than hyperdiploidy, secondary chromosomal translocations, and gene mutations occur in subclones of MM cells [2,6,15] (Figure 1).

Over the last two decades, the introduction of autologous hematopoietic stem cell transplantation (ASCT), immunomodulatory drugs (IMiDs), proteasome inhibitors (PIs), and an anti-CD38 monoclonal antibody have greatly improved the outcomes of patients with MM [16]. However, patient overall survival (OS) varies from less than 2 years to more than 10 years [1]. Chromosomal aberrations such as t(4;14), t(14;16), t(14;20), gain/amplification of chromosome arm 1q21 (1q21+), and deletion of chromosome arm 17p (del(17p)) are associated with a worse prognosis in patients with MM [1,17,18,19]. 

The chromosomal aberration 1q21+ is the most common prognostic chromosomal abnormality that occurs in about 40% of patients with newly diagnosed MM (NDMM). It was first reported as an adverse cytogenetic aberration for MM in the Total Therapy 2 (TT2) trial (tandem ASCT +/− thalidomide) [20,21,22,23]. NDMM patients with 1q21+ showed inferior event-free survival (EFS) and OS compared with those lacking 1q21+. Adding thalidomide improved EFS in patients lacking 1q21+, but not in those with 1q21+. Since then, PIs and newer IMiDs have been introduced for the treatment of MM. However, many studies have found the adverse impact of 1q21+ on survivals, and the prognostic value of 1q21+ has been investigated in detail [24,25,26,27,28,29,30,31,32,33]. 

Trisomy of chromosome 1, tandem duplications of 1q21, and jumping whole-arm translocations of 1q (JT1q) are some of the ways in which 1q21+ occurs, suggesting that patients with 1q21+ are heterogeneous at the genomic level [34,35,36]. The de-condensation of heterochromatin in the 1q12 region may cause chromosomal instability resulting in JT1q and segmental duplications of 1q21 which lies adjacent to 1q12 [34,35,36]. 

It is suggested that genes located in the 1q21 amplicon, including *CKS1B*, *PSMD4, IL6R, ADAR, MCL1*, and others, are associated with tumor proliferation and/or drug sensitivity because of upregulation of the expressed genes resulting from the increased gene dosage in MM cells with 1q21+ [37,38,39,40,41,42,43,44,45]. It has been demonstrated by experimental cellular models, and/ or correlation analysis of gene expression levels and prognosis in a large patient cohort.

In this review, we describe the current knowledge on the clinical features, prognostic value, and amplification mode of 1q21+, as well as the possible responsible genes located on 1q21. This can provide clues for the effective therapeutic approach to MM patients with 1q21+ to further improve outcomes of patients with MM. 

## 2. Clinicopathological Features of 1q21+ in MM

### 2.1. Incidence, Detection, and Clinical Features of 1q21+ in MM

The incidence of 1q21+ increases from MGUS to relapsed/ refractory MM (RRMM) [23]. It is detected in ~20% of MGUS, 30–50% of SMM and NDMM, and 50–80% of RRMM [23,46,47,48,49,50]. SMM patients with 1q21+ progress to MM more frequently than those without 1q21+ [22,50]. It is also found in about 70% of patients with primary plasma cell leukemia [51], and in about 90% of patients with a rare morphologic variant of MM, anaplastic myeloma [52]. 1q21+ is likely to be associated with the disease progression and drug resistance of MM, and the aggressive phenotype of plasma cell disorders.

Detection of 1q21+ by interphase fluorescence in situ hybridization (FISH) is generally performed on plasma cells purified with CD138-microbeads (CD138-FISH) from bone marrow (BM) samples [53], or by combining FISH with the patient-specific cytoplasmic immunoglobulin light chain staining with amino-methyl-coumarin-acetic acid (AMCA) on fixed mononuclear cells from the BM (cIg-FISH) [54]. The FISH probe DNA for 1q21 is usually from a BAC (bacterial artificial chromosome) clone, RP11-307C12, which is located in the most amplified region of 1q21 containing the *CKS1B* gene [22,23,37]. 1q21+ can also be evaluated using array comparative genomic hybridization (aCGH), single-nucleotide polymorphism (SNP)-based arrays, multiplex ligation dependent probe amplification (MLPA) system, and next-generation sequencing (NGS) on DNA from MM cells purified with CD138-microbeads [28,31,37,42,55,56,57]. The incidence of 1q21+ found using arrays could be less when the proportion of cells with 1q21+ is low in MM cells.

In patients with NDMM, the presence of 1q21+ is likely to be associated with IgA type, high levels of serum lactate dehydrogenase and β2-microglobulin, low level of serum albumin, hypercalcemia, anemia, lower level of platelets, higher tumor burden, low frequency of international staging system (ISS)-1, and high frequency of ISS-3 [23,27,32,58,59]. The incidence of lytic bone lesions is similar between patients with 1q21+ and those without 1q21+ [23,27,32]. Chromosomal aberrations such as G-band abnormalities, t(4;14), t(14;16), t(14;20), deletion of 1p (del(1p)), and deletion of 13q (del(13q)) are likely to be frequently observed in patients with 1q21+ compared with those lacking 1q21+ [23,27,32,58,59]. In contrast, t(11;14) is infrequent in cases of 1q21+ [23,32]. 1q21+ is not likely to be associated with del(17p). Hyperdiploidy is also not associated with 1q21+, however, 1q21+ has been reported to be less frequent in hyperdiploid patients with trisomy of chromosome 11, which is a subgroup with upregulation of the *CCND1* gene [57]. 

Since 1q21+ occurs as a secondary event in myeloma subclones, the proportion of cells with 1q21+ in MM cells varies among patients. In a study by An et al. [47], the incidence of MM cells with 1q21+ determined using cIg-FISH was 46% (clone size, 0–10%), 4% (10.5–20%), 12% (20.5–50%), 37% (≥50.5%) in patients with NDMM [46], which is consistent with a previous report [23]. The cut-off value to designate a patient as ‘1q21+ positive’ by interphase FISH varies from less than 5% to 30% among reports. An et al. reported that the cut-off of 20% was the best possible value for predicting poor outcomes using receiver-operating characteristics analysis in patients with 1q21+ receiving either thalidomide- or bortezomib-based treatments [47].

### 2.2. Prognostic Implications of 1q21+ in MM

The prognostic value of a parameter depends on the treatment regimens, cohorts, and newly identified prognostic markers. 1q21+ has been suggested as a poor prognosis factor in a number of studies in NDMM [24,25,26,27,28,29,30,31,32,33]. The first report on the prognostic analysis of 1q21+ in a cohort of the TT2 study showed that 1q21+ was an independent poor prognostic predictor for EFS and OS (Table 1), and that adding thalidomide did not improve the EFS and OS in patients with 1q21+ [21,23]. In a study by Avet-Loiseau et al. with 520 NDMM patients younger than 66 years of age from the French IFM99-02 and 99-04 trials [60], with none of the patients receiving PIs and/or IMiDs in the first-line induction treatment, 1q21+ was independently associated with poor OS but not with progression-free survival (PFS). In another study by Shah et al. with 1905 NDMM patients from the UK Myeloma IX and Myeloma XI studies [28], 1q21+ was found to be independently associated with poor PFS and OS in both studies using multivariate analysis including adverse *IGH* translocations (t(4;14), t(14;16), or t(14;20)), del(17p), and the advanced ISS stage. In a retrospective study by Schmidt et al. with 201 consecutive NDMM patients receiving induction with VRd (bortezomib + lenalidomide + dexamethasone) in their institute [30], patients with 1q21+ showed independently poor PFS and OS compared with those without 1q21+. Interestingly, the best response to VRd induction was better in patients with 1q21+; those with 1q21+ were more likely to achieve a response ≥ very good partial response (VGPR) compared with patients lacking 1q21+ (75% vs. 59%, *p* = 0.02). More recently, in a large retrospective study by Abdallah et al. with 1376 Mayo Clinic patients, diagnosed with MM from 2005 to 2018, who received induction with PIs-, or IMiDs-, or PI + IMiD- based therapy [32], 1q+ (they investigated the prognostic effects of gain/amplification of 1q22 which was usually co-amplified with 1q21) was independently associated with poor OS, irrespective of whether ASCT was done or not. These studies suggest that at diagnosis, 1q21+ seems to be an independent adverse prognosis marker for OS, even in the era when PIs and/ or IMiDs are used. The data on prognostic significance of 1q21+ in patients treated with the first-line daratumumab-combo is currently limited.

1q21+ at relapse is also associated with a shortened post-relapse survival. In the TT2 study, patients with 1q21+ at relapse showed inferior post-relapse survival compared with those lacking 1q21+ at relapse (5-year rate, 15% vs. 53%, *p*= 0.02) [23]. In a study by Chang et al. in 85 RRMM patients treated with bortezomib-based therapy at relapse [60], who did not receive bortezomib previously, patients with 1q21+ had a shorter OS (median, 5.3 months vs. 24 months, *p* < 0.001) and PFS (median, 2.3 months vs. 7.3 months, *p* = 0.003) compared with those without 1q21+. Multivariate analysis including del(13q), t(4;14), del(17p), del(1p21), and high serum β2-microglobulin level (>3.5 mg/L) indicated that 1q21+ was an independent adverse factor for both OS and PFS. In a study by Klein et al. with 92 RRMM patients treated with lenalidomide and dexamethasone as salvage therapy [61], 1q21+ at relapse was independently associated with a shorter post-relapse survival. More recently, in a study by Mohan et al. with 81 RRMM patients who were previously exposed to both PIs and IMiDs and treated with daratumumab monotherapy or in combination with other drugs [62], patients with 1q21+ at diagnosis or first visit to their institute (University of Arkansas for medical Sciences, UAMS) showed inferior PFS and OS compared with those without 1q21+ (median PFS, 0.5 years vs. 2.1 years, *p* = 0.0004; median OS, 0.9 years vs. not reached, *p* = 0.002). As the incidence of 1q21+ is increased during disease progression, showing worse prognosis at any time, 1q21+ seems to be deeply related with poor OS in MM.

1q21+ can be divided into 2 categories according to the increased levels of the copy number of 1q21 [23,25,28,29,30,31,63,64]. In studies using interphase FISH for 1q21+ detection, the patients are usually defined as those having amplification of 1q21 (amp(1q21)) when ≥10% (or 20%) of MM cells harbor at least 4 copies of 1q21, and the remaining patients with 1q21+ are defined as those having gain of 1q21 (gain(1q21)), however, a uniform definition for amp(1q21) is absent. Gain(1q21) and amp(1q21) are seen in about 20–30% and 5–20% of patients with NDMM, and in about 20–30% and 30–45% of patients with RRMM, respectively [23,25,63]. The number of copies of 1q21 and the proportion of cells with 1q21+ in MM cells also show a trend, increasing at relapse compared with diagnosis [23]. The increased copy number of 1q21 is likely to be associated with higher proliferation ability.

It has been suggested that the adverse impact of 1q21+ on survival was profound in patients with amp(1q21) compared to those with gain(1q21). In the TT2 study [23], NDMM patients with amp(1q21) showed an initially more aggressive clinical course than those with gain(1q21), but EFS and OS were similar between patients with amp(1q21) and gain(1q21). In contrast, RRMM patients with amp(1q21) at relapse showed a trend of a shorter post-relapse survival compared with those with gain(1q21) (5-year post relapse survival rate, amp(1q21) vs. gain(1q21): 0% vs. 32%, *p* = 0.06). In a study by Neben et al. from the HOVON-65/GMMG-HD4 trial [25], NDMM patients with amp(1q21) showed inferior OS compared to those with gain(1q21) (Hazard ratio (HR) for PFS and OS; no 1q21+ vs. gain(1q21) and no 1q21+ vs. amp(1q21): 1.65/2.48 and 1.66/3.95, *p* = 0.001/0.006 and 0.031/<0.001, respectively). In a study by Walker et al. with 863 NDMM patients with an age < 75 years [31], patients with amp(1q21), as defined by NGS, showed inferior PFS and OS compared to those with gain(1q21) (estimated rates at 18-months, gain(1q21) vs. amp(1q21); PFS/OS: 71/88 vs. 60/73%, *p* = 0.06/0.08, respectively). They also found that Kaplan-Meier curves for OS were similar between patients with gain(1q21) and amp(1q21) until around 12 months after initiation of therapy, but clear separation in the curves was seen after 12 months. More recently, in a study by D’Agostino et al. from the Forte trial including 474 NDMM patients treated with carfilzomib-based induction followed by ASCT [65], patients with amp(1q21) showed inferior PFS and OS compared to those with gain(1q21) (HR for PFS and OS: 1.8 and 3.1, *p* = 0.004 and <0.001, respectively). In a study by Ziccheddu et al. with 42 patients refractory to both PIs and IMiDs [66], where 83% of patients expired within 1000 days of sampling, amp(1q21) was found in 45% of patients, and amp(1q21), but not gain(1q21), showed a significantly poor PFS and a trend towards worse OS compared to those with 2 or 3 copies of 1q21. On the other hand, patients with bi-allelic loss of the wild-type *TP53* gene, which was detected in 15% of patients in the cohort, showed similar PFS and OS compared to those with at least one copy of the wild-type *TP53* gene. 

Co-existence of adverse chromosomal abnormalities such as t(4;14), t(14;16), del(1p), and del(17p), worsened the prognosis in patients with 1q21+ [67,68]. Hyperdiploid patients with 1q21+ show inferior PFS compared with those without 1q21+ [69]. In a study by Schmidt et al., with 201 NDMM patients who were uniformly treated with VRd induction, among the patients lacking adverse cytogenetics (including t(4;14), t(14;16), and del(17p)) patients with gain(1q21) showed similar PFS and OS as patients with 2 copies of 1q21, while patients with amp(1q21) showed worse prognosis than those with 2 or 3 copies of 1q21 [30]. More recently, in a study by Locher et al. with 794 NDMM patients [64], in which >95% were treated with an induction of PIs and/or IMiDs and 44% underwent a front-line ASCT, among patients with clonal gain of chromosome 11 and without any primary *IGH* translocations, patients lacking 1q21+ showed a markedly long median OS of more than 9 years. This subgroup is likely to correspond to patients with hyperdiploidy lacking 1q21+. 1q21+ seems to be a useful predictor that, in combination with other predictive cytogenetic abnormalities, can discriminate patients with a clearly favorable prognosis. This can help guide the patients to treatments. 

In other recent reports, prognostic relevance of 1q21+ has been reported for lenalidomide maintenance for patients with NDMM [70], addition of ixazomib with Rd for RRMM [71], addition of elotuzumab with Rd for RRMM [72], and addition of isatuximab with pomalidomide and dexamethasone (Pd) for RRMM [73]. In a study by Jackson et al. from the Myeloma XI trial [70], it was shown that lenalidomide maintenance improved the PFS compared with placebo in patients with 1q21+ (HR, 0.46; 95% CI, 0·33–0·62). HR for PFS of lenalidomide to placebo in patients with 1q21+ seemed to be similar to that in patients without 1q21+ (HR; 1q21+: 0.46 and no 1q21+: 0.39). In the TOUMALIME-MM1 study (ixazomib + lenalidomide + dexamethasone (IRd) vs. Rd) [71], IRd showed a tendency to improve PFS compared to Rd in RRMM patients with 1q21+ and lacking t(4;14), t(14;16), and del(17p) (IRd vs. Rd, HR, 0.78; 95% CI, 0.49–1.2). In the ELOQUENT-2 study (elotuzumab + lenalidomide + dexamethasone (ERd) vs. Rd) [72], ERd showed a superior PFS compared to Rd among patients with 1q21+ (ERd vs. Rd, HR, 0.75; 95% CI, 0.56–0.99). These drugs can be administrated for a relatively long time, which might be associated with contribution to PFS prolongation. More recently, in a study by Richardson et al. from the ICARIA-MM study (isatuximab + pomalidomide + dexamethasone (Isa-Pd) vs. Pd) [73], in 1q21+ RRMM patients lacking another adverse cytogenetics (t(4;14), t(14;16), and del(17p)), Isa-Pd showed a superior PFS compared with Pd (HR 0.49, 95% confidence interval (CI) 0.3–0.9). In patients without 1q21+, t(4;14), t(14;16), and/or del(17p), Isa-Pd showed a tendency for a superior PFS compared with Pd (HR 0.63, 95% CI 0.3–1.3). Isa-Pd might be more beneficial for 1q21+ RRMM patients lacking another adverse cytogenetics.

In addition to the presence of adverse cytogenetics, achieving a minimal residual disease (MRD)-negativity has become an important prognostic factor in MM [74]. Interestingly, even though 1q21 + adversely affects survival outcomes, when patients are treated with induction of PI + IMiDs, the proportion of patients who achieve ≥CR and ≥VGPR in patients with 1q21+ have been reported to be similar or better compared to those in patients lacking 1q21+ [30,32]. However, the data regarding the effect of 1q21+ on achieving MRD-negativity, and the relationship between MRD-negativity and prognosis in patients with 1q21+ are currently lacking.

### 2.3. Amplification Patterns of 1q21+ in MM

**Figure 2:**
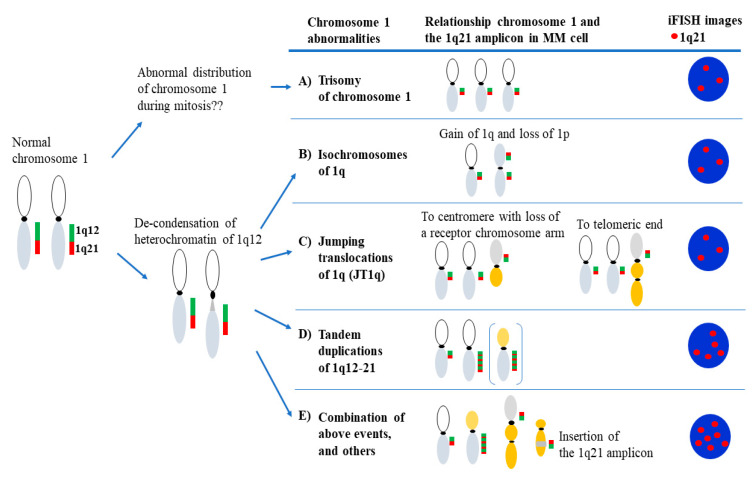
Schematic images of chromosome 1 aberrations and the 1q21 amplicon in multiple myeloma patients with 1q21+. Gain/amplification of 1q21 (1q21+) is resulting from trisomy of chromosome 1 (**A**), and structural changes of chromosome 1 due to chromosomal instability of 1q12 (**B**–**E**). Green and red bar indicate the 1q12 and 1q21 regions, respectively. iFISH, interphase fluorescent in situ hybridization.

According to the analysis of the metaphase spread of patient MM cells, 1q21+ is mostly caused a result of trisomy of chromosome 1, isochromosomes of 1q, JT1q, and tandem duplications of 1q21 (dup(1q21)) [34,35,36,75,76]. (Figure 2).

In a series of studies by Sawyer et al., 1q21+ is suggested to be caused by a chromosomal instability of 1q12 due to the de-condensation of heterochromatin region in the chromosome 1q pericentromeric region, and breakage of the centromere/pericentromeric region of chromosome 1q [34]. G-band analysis of patient MM cells revealed that aberrations of chromosome 1q frequently involved 1q12 triploids, multibranched chromosomes and isochromosomes of 1q, and JT1q, resulting in 1q21+ [34,75]. Combination of spectrum karyotyping and metaphase FISH with probes for 1q12 and 1q21 revealed that segmental duplications of chromosome 1q12–21 occurred on homologous and/or non-homologous chromosomes [35]. Segmental duplications of chromosome 1q12–21 can occur over 8 times, and the amplicon can span the region of 1q12-1q25 [36], corresponding with the most amplified region of around 10-15 Mb detected by aCGH which contains more than 500 genes [37]. JT1q causes not only whole-arm-level copy gains of chromosome 1q, but also whole-arm-level loss of the translocation receptor chromosomes of JT1q when whole-arms of chromosome 1q translocate to the centromeric region of certain chromosomes (Figure 2). Because of JT1q, genes mapped to 1q are ”mildly” amplified, and genes mapped to arms of the receptor chromosome can be decreased. On the other hand, dup(1q21) results in ”highly” increased copy numbers of gene sets in the 1q21 amplicon, and loss of gene(s) at the loci of dispersed insertions of the 1q21 amplicon. The phenotype of MM cells with 1q21+, including chromosomal instability of 1q12, can be inherited by daughter cells. MM cells with 1q21+ might become more malignant over time via chromosomal instability of 1q12 [36]. The prognostic value of 1q21+ is usually investigated by using the copy number of 1q21 defined by interphase FISH or other methods. The relevance of 1q21+ for an aggressive phenotype of MM might be a surrogate for chromosomal instability rather than increased copy number of 1q21.

### 2.4. Possible Responsible Genes Located on Chromosome Band 1q21 in MM

**Figure 3:**
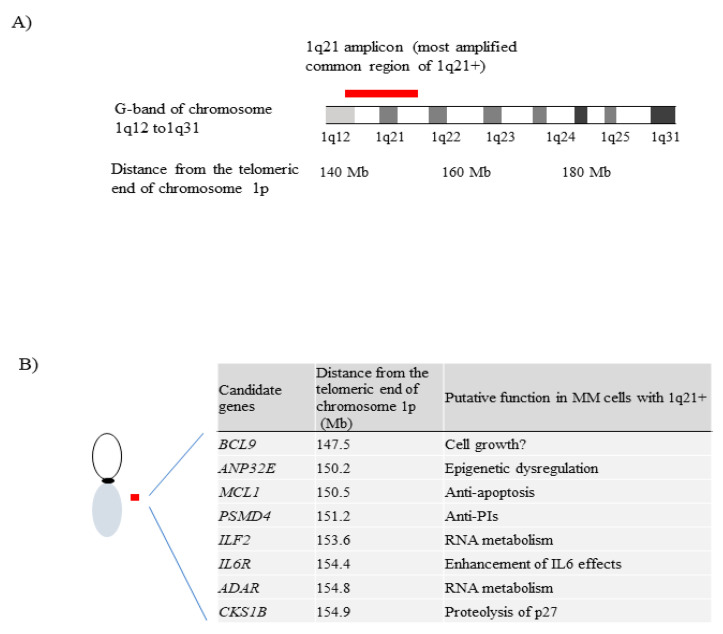
The 1q21 amplicon and possible responsible genes at 1q21 in multiple myeloma. Relationship of the segmental duplication locus and G-band of chromosome 1q12–21 (**A**). Possible responsible genes in the 1q21 amplicon and putative function of those genes in multiple myeloma (MM) cells with 1q21+ (**B**). Red bar indicates the 1q21 amplicon. BCL9, B-cell CLL/lymphoma 9; ANP32E, acidic nuclear phosphoprotein 32 family member E; MCL1, myeloid leukemia cell differentiation protein 1; PSMD4, proteasome 26S subunit, non-ATPase 4; ILF2, interleukin enhancer binding factor 2; IL6R, interleukin 6 receptor; ADAR, adenosine deaminase RNA specific; CKS1B, CDC28 protein kinase regulatory subunit 1B.

There are over 500 genes located on chromosome 1q21-25, which is the commonly amplified region in patients with 1q21+ [36,37,42]. In MM cells, not all genes present on the 1q21 amplicon are expressed, but the expressed genes should be co-upregulated due to the increased gene dosage in cells with 1q21+. Among those genes in the 1q21 amplicon, several genes have been reported to be associated with aggressive phenotypes of MM (Figure 3). Aggressive phenotypes were demonstrated by using cellular experimental models and/or analyzing the correlation between prognosis and gene expression levels. 

*CKS1B*, CDC28 protein kinase regulatory subunit 1B, was first reported as a possible candidate gene at 1q21 by Zhan et al. [39]. In MM cells from patients, expression levels of CKS1B protein were increased according to the elevated levels of the *CKS1B* mRNA, showing an inversed correlation with expression level of p27^KIP1^ protein, but not p27^KIP1^ mRNA. The p27^KIP1^ protein negatively regulates the G1 to S transition of cell cycle. In the human myeloma cell lines (JJN3, OCI-MY5, and XG1), overexpressing *CKS1B*, silencing of *CKS1B* expression was associated with the upregulation of p27^KIP1^ which resulted in inhibition of the cell growth. The *CKS1B* gene is known to code for a co-factor for the SKP2-dependent ubiquitination of p27^Kip1^. Overexpression of *CKS1B* is likely to be associated with the 1q21+ [22], and promoted cell proliferation partially due to proteolysis of p27^KIP1^ protein in MM cells with 1q21+. 

*PSMD4*, proteasome 26S subunit, non-ATPase 4, was also reported as a possible responsible gene at this locus [40,77]. Shaughnessy et al. have developed gene expression profiling (GEP)-based risk prediction models, designated GEP70 and GEP80 [40,77]. GEP70 was developed to predict patients with early disease-related death that constituted around 15% of NDMM patients in the cohort (5-year EFS/OS, high-risk vs. low-risk: 18/28% vs. 60/78%, *p* < 0.001, both) [77]. Of the 70 highly survival-discriminatory genes in GEP70, 9 of the 51 up-regulated genes mapped to chromosome 1q, and 9 of the 19 downregulated genes mapped to chromosome 1p [77], suggesting that the GEP70 signature is relevant to a subtype having both 1q21+ and del(1p). GEP80 was developed based on GEP alterations 48 h after bortezomib test dosing and linked to poor PFS in patients with NDMM; it consists of 80 survival-discriminatory genes [40]. These 80 genes primarily affected the protein ubiquitination pathway. The *PSMD4* gene was one of two up-regulated genes common to both GEP70 and GEP80 models. *PSMD4* expression levels were associated with the copy number of 1q21, and higher *PSMD4* expression levels effected adverse clinical outcomes [40]. PSMD4 is an essential subunit of 19S proteasome complex, which is a component of the 26S proteasome, associated with intracellular proteolysis. Higher expression of *PSMD4* resulting from amp(1q21) may be associated with adverse prognosis due to reduction of the anti-myeloma effect of PIs. More recently, in a study by Misiewicz-Krzeminska et al., 174 patients with NDMM from the Spanish PETHEMA/ GEM2012 study were treated with VRd induction followed by ASCT and VRD consolidation [78]. A higher protein expression of PSMD4 was found to be associated with a poor outcome (median time-to progression months, PSMD4 protein low vs. high: 48 vs. not reached, *p* = 0.02).

In a study by Walker et al., based on the 1q21 copies and expression quartile analysis, *ANP32E* (acidic nuclear phosphoprotein 32 family member E) was identified as a gene of prognostic importance at 1q21 [42]. ANP32E, a specific H2A.Z histone chaperone [79], belongs to a family of proteins with leucine-rich repeats [80]. ANP32E is thought to remove H2A.Z from chromatin to regulate gene expression [79]. In recent studies, ANP32E has been shown to contribute to variable cancers such as breast, thyroid, and pancreatic cancers [81,82,83]. Biological function of ANP32E in MM has not been reported, but overexpression of *ANP32E*, resulting from amp(1q21), may have a pathogenic role in MM as reported for other cancers.

*IL6R*, interleukin 6 receptor, is also located in the 1q21 amplicon. *IL6R* is a gene encoding a subunit of IL6 receptor complex. IL6 is a pleiotropic cytokine produced by a variety of cells including MM cells. It is a pivotal cytokine for survival and proliferation of MM cells, and high expression of IL6R can increase sensitivity to IL6 [84,85]. Expression level of IL6R mRNA was associated with the 1q21 copies [42,86]. In a study by Stephens et al. using MM patient samples [41], combination of 1q21+ and the SNP rs2228145 minor allele C, which lies within coding region of the IL6R transmembrane-domain, was associated with high concentration of serum soluble IL6R (sIL6R), associated with poor prognosis. High expression of IL6R is likely to be associated with poor prognosis via upregulation of sensitivity to IL6, and the SNP rs2228145 in *IL6R* may affect increased production of sIL6R and prognosis.

The gene encoding MCL1, a member of the BCL2 family anti-apoptotic protein, is also located in the 1q21 amplicon. MCL1 has been reported to be essential for normal plasma cell survival [87]. Survival of MM cells also has been reported to be dependent on MCL1, or BCL2 or both protein [88]. In many cancers, overexpression of MCL1 is frequently observed, and associated with resistance to cytotoxic therapies. MCL1 inhibitors have been developed and are currently investigated in clinical trials in many cancers including MM. In a study by Slomp et al. using 47 NDMM patient samples [43], MM cells from patients with 1q21+ were more sensitive to MCL1 inhibition compared with those lacking 1q21+. MCL1 inhibitors might be more effective in MM patients with 1q21+ compared to those lacking 1q21+ and without BCL2 expression.

*ADAR*, adenosine deaminase acting on RNA, is located adjacent to *IL6R* in the 1q21 amplicon. ADAR is an RNA-binding protein and associated with RNA-editing by deamination converting adenosine to inosine. Adenosine-to-inosine RNA editing makes the RNA unstable and has been suggested to be involved with cancer development. Expression of p150 isoform of ADAR is induced by inflammatory cytokines such as IL6. In a study by Lazzari et al. using the MMRF CoMMpass database [86], patients with higher expression of *ADAR* showed poor prognosis, and expression of *ADAR* was higher in patients with 1q21 compared with those lacking 1q21+. In addition, expression of *ADAR* enhances *Alu*-dependent RNA editing and transcriptional activity of GLI1 (glioma-associated oncogene 1) that is a positive effector of Hedgehog signaling and promoted resistance to lenalidomide of MM cells. Silencing of *ADAR* expression reduced regeneration of high-risk MM tumors in serially transplantable patient-derived mice xenografts. High expression of *ADAR* caused by 1q21+ and IL6 may be involved with the pathogenesis of MM.

*ILF2*, interleukin enhancer-binding factor 2, is also related with RNA metabolism and located in the 1q21 amplicon. *ILF2* is a gene encoding the subunit of a transcription factor, NFAT (Nuclear factor of activated T-cells). NFAT is a heterodimer of 45 kDa (Nuclear factor (NF) 45) and 90 kDa proteins (NF90), of which NF45 is a product of the *ILF2* gene. NFAT has been reported to play essential roles in mitosis through regulation of mitotic mRNAs [89]. Marchesini et al. found that overexpression of ILF2 promoted tolerance of genomic instability and resistance to DNA damaging drugs such as melphalan in MM cells with 1q21+ [90]. High expression of *ILF2* might be a mechanism of resistance to conventional treatments such as alkylators of melphalan and cyclophosphamide in patients with amp(1q21). 

Since the whole-arm-level gains of chromosome 1q occur in MM cells with 1q21+, genes located on chromosome 1q other than the 1q21 amplicon can be ”mildly” upregulated. The *CD46* and *CD55* genes located at 1q32 [91,92] negatively regulate CDC (complement-dependent cytotoxicity) of monoclonal antibodies (mAb) such as daratumumab. Up-regulation of these molecules may in part account for resistance to mAb therapy having CDC. The *C1orf35* (chromosome 1 open reading frame 35) gene at 1q42, which is thought to upregulate the expression of *MYC*, has also been reported to be associated with the aggressive phenotype of MM with 1q21+ [93]. 

## 3. Conclusions and Future Directions

The chromosomal aberration 1q21+ is found in about half of the patients with NDMM and is likely to be an independent poor prognosis factor for OS, even in the new drug era of PIs and IMiDs. The incidence of 1q21+ and the copy number of 1q21 in MM cells increase during disease progression, showing a constant poor prognosis, suggesting that 1q21+ is closely associated with disease progression and drug resistance in MM. Co-occurrence of other adverse chromosomal abnormalities with 1q21+ are likely to be associated with a worse prognosis, and amp(1q21) is also likely to have a worse prognosis than gain(1q21). Even in the era of novel agents 1q21+ still portends a poor prognosis as compared to negative patients.

1q21+ may be caused by trisomy of chromosome 1, gains of chromosome arm 1q related with JT1q, and segmental duplications of 1q12-25. The latter 2 events are thought to result from chromosomal instability of 1q12, which can also introduce arm-level and/or partial losses in associated receptor chromosomes, resulting in reduced expression of genes on those receptor chromosomes. This simultaneous, multiple chromosomal arm events result in chromosomal instability in MM cells, which may cause progressive complex cytogenetic changes over time, including increased copy number of 1q21. This may be a mechanism accounting in part for the malignant phenotype of MM cells with 1q21+.

Along with the chromosomal instability in cells with 1q21+, highly increased expression of genes in the 1q21 amplicon are also likely to contribute to the development of the malignant phenotype of MM cells with 1q21+. Among over 500 genes located in the 1q21 amplicon, several are reported to be associated with the malignant phenotype of MM cells. The precise roles of these genes in MM cells have not been completely elucidated, but their putative function is heterogenous. For example, *CKS1B* is associated with the promotion of G1-S transition, *PSMD4* is associated with resistance to PIs, *ANP32E* is associated with epigenetic dysregulation, *IL6R* is associated with promotion of cell growth and survival by IL6, and *ADAR* and *ILF2* are likely to be associated with RNA metabolism. The amplification of the 1q21 region results in the simultaneous enhancement of the function of various genes in the amplicon, leading to more aggressive phenotypes of cells with 1q21+, which might, in part, account for the resistance to different treatment regimens. 

Targeting candidate gene(s) in the 1q21 amplicon might be beneficial, especially for patients with 1q21+ who do not benefit from currently available therapies. However, once 1q21+ occurs in MM cells, the cells become more resistant to therapies over time. Therefore, early intervention with more potent investigational therapies might be beneficial. Further clarification on the mechanisms that cause 1q21+ and the roles of genes in the 1q21 amplicon may provide potent novel targets for the development of effective treatments for MM patients with 1q21+.

## Figures and Tables

**Table 1:** Survival impacts of 1q21+ in newly diagnosed patients with multiple myeloma.

Trial or Institution (Publication Year)	Patient Number	Cohort Type	First-Line Treatment	1q21+ vs. no 1q21+		Reference
				PFS	OS	
Total Therapy 2 (2006)	479	RCT	ASCT ± thalidomide	HR 1.8, *p* < 0.001 (event-free survival)	HR 1.7, *p* = 0.005	Hanamura et al. [23]
IFM99-02 and 99-04 (2012)	520	RCT	VAD-ASCT	HR NA, *p* > 0.05	HR 1.5, *p* = 0.006	Avet-Loiseau et al. [60]
HOVON-65/GMMG-HD4 (2012)	354	RCT	VAD or PAD-ASCT	HR 1.5, *p* = 0.01	HR 1.6, *p* = 0.04	Neben et al. [25]
Myeloma IX and Myeloma XI (2017)	1905	RCT	Myeloma IX, CVAD or MP or CTD; Myeloma XI, CTD or CRD	HR 1.5, *p* < 0.001	HR 1.6, *p* < 0.001	Shah et al. [28]
Emory University (2019)	201	Retrospective	VRD	HR 1.9, *p* = 0.002	HR 2.6, *p* = 0.002	Schmidt et al. [30]
Mayo Clinic (2020)	1376	Retrospective	PI based 36%	19 vs. 27 mo, *p* < 0.001	HR 1.5, *p* < 0.001	Abdallah et al. [32]
			IMiDs based 35%	(time to next treetment)		
			PI+IMiDs based 28%			

RCT, randomized controlled trial; ASCT, autologous hematopoietic stem cell transplantation; VAD, vincristine, adriamycin, dexamethasone; PAD, bortezomib, adriamycin, dexamethasone; CVAD, cyclophosphamide, vincristine, adriamycin, dexamethasone; MP, melphalan, prednisone; CTD, cyclophosphamide, thalidomide, dexamethasone; CRD, cyclophosphamide, lenalidomide, dexamethasone; VRD, bortezomib, lenalidomide, dexamethasone; PI, proteasome inhibitor; IMiDs, immunomodulatory drugs; PFS, progression-free survival; OS, overall survival; HR, hazard ratio; NA, not available; mo, month.
